# Craniomandibular transverse tomographic evaluation after anterior open bite orthodontic treatment with miniplates anchorage

**DOI:** 10.1186/s40510-024-00519-1

**Published:** 2024-05-27

**Authors:** Enio Vitor de Mesquita, Fernanda Meloti, Ertty Silva, Mauricio de Almeida Cardoso, Tien-Li An, Monikelly do Carmo Chagas Nascimento

**Affiliations:** 1 Division of Orthodontics, Faculdade São Leopoldo Mandic, Instituto de Pesquisas São Leopoldo Mandic, Campinas, São Paulo, SP Brazil; 2 Department of Dentistry, University of Brasilia, School of Health Sciences, Distrito Federal, Brasília, Brazil; 3 Division of Oral Radiology, Faculdade São Leopoldo Mandic, Instituto de Pesquisas São Leopoldo Mandic, R. Dr. José Rocha Junqueira, 13-Pte. Preta, Campinas, São Paulo, SP 13045-755 Brazil

**Keywords:** Transverse discrepancy, Cone-beam computed tomography, Miniplates, Anterior open bite

## Abstract

**Background:**

Skeletal anterior open bite (SAOB) represents one of the most complex and challenging malocclusions in orthodontics. Orthodontic treatment supported by miniplates enable to reduce the need for orthognathic surgery. Transverse dimension may be affected by intrusion biomechanics. This study aims to assess transverse bone alterations in patients with SAOB who underwent orthodontic treatment with absolute anchorage using four miniplates.

**Methods:**

A total of 32 patients of both sexes, with an average age of 33.8 years, diagnosed with SAOB and treated orthodontically with four miniplates (one in each hemiarch), were selected for this study. Tomographic examinations were performed before (T1) and after (T2) orthodontic treatment. Linear measurements (width of the maxillary base, maxillary alveolar, maxillary root, maxillary dental cusp, mandibular alveolar) and angular measurements (maxillary intermolar angle) were assessed in these images. The Shapiro-Wilks normality tests were applied to verify data distribution, and the paired t-test was used to compare the initial and final measures obtained.

**Results:**

Among the evaluated parameters, the maxillary alveolar width, maxillary dental cusp width, mandibular alveolar cusp width, and intermolar angle showed statistically significant differences between T1 and T2 (*p* < 0.05). However, maxillary base and maxillary root widths showed no significant difference (*p* > 0.05).

**Conclusions:**

Intrusion and distalization with miniplates in SAOB therapy may lead to significant expansive changes, due to molars cusps width and buccal inclination increase restricted at the alveolar level.

## Background

For a long time, the diagnosis of the transverse dimension was made through orthodontic models or through a clinical evaluation, directly in the patient’s mouth, leading to subjective assessments. Commonly used radiographs, such as lateral and panoramic teleradiography, lacked three-dimensional analysis (sagittal, transverse and vertical). On the other hand, frontal or posteroanterior radiographs, which were occasionally used, made it difficult to interpret the images, due to the large overlapping of structures [1, 2].

With the advent of cone beam computed tomography (CBCT), it was possible to evaluate bone and dental structures in three dimensions, which enabled a broad interpretation of the images obtained, due to the lack of overlapping structures neighboring the region of interest. Thus, its increasing use in Dentistry can be seen, with decreasing costs and radiation doses [3]. In addition, CBCT enables the use of new methods of craniofacial diagnosis, as 3D rendering allows detailed analysis of skull and facial morphology, supporting innovative orthodontic treatments and clinical practices [4].

Orthodontic mechanics, combined with absolute skeletal anchorage provided by miniplates, supported by 3D diagnosis, yield superior results compared to skeletal anchorage with mini-implants, considered relative anchorage [5]. Miniplates allow application of greater magnitude forces, as they are fixed in the thick and dense cortical bone, with up to three osseointegrated screws and away from the dental roots [5, 6]. Simultaneously, they permit tooth movements in all three spatial planes, with lower biological cost [6, 7]. Additionally, miniplate-supported intrusion mechanics may result in buccal tipping and dentoalveolar expansion, which may be desirable or undesirable depending on treatment goals. Overall, orthodontic mechanics with miniplates exhibit high success rates [6], particularly in cases requiring significant force application involving multiple teeth [9].

In Orthodontics, skeletal anterior open bite (SAOB) represents one of the most complex and challenging malocclusions. Due to its multifactorial nature, various treatments have been proposed, often with high recurrence rates [10]. The advent of skeletal anchorage and CBCT has improved diagnosis accuracy and treatment planning predictability, reducing the need for orthognathic surgery, previously considered the gold standard treatment [5, 11]. Orthodontic mechanics combined with increased skeletal anchorage using miniplates is indicated for SAOB treatment, facilitating occlusal plane correction and achieving muscular, skeletal, and dental balance [12].

Studies using CBCT to assess intrusion mechanics have predominantly focused on assessing the intrusion effect, whereas research on arch width changes associated with skeletal anchorage use is limited in the literature. Considering that intrusion biomechanics may impact the transverse dimension, evaluating these changes is crucial for ensuring predictable control during orthodontic planning in the management of SAOB patients. Thus, the present study aims to evaluate the transverse alterations occurring in the first molars, elucidating the skeletal and dental effects following the respective treatment.

## Material and methods

### Sample selection

This observational and retrospective study was submitted and approved by the local Research Ethics Committee, under number 4,805,732. CBCT DICOM (Digital Imaging and Communications in Medicine) files that belonged to a private clinic database were used. CT scans were acquired using the i-CAT device (Imaging Sciences International, Hatfield, PA, United States) with the following parameters: Field of View (FOV) 23 × 17 cm, 0.4 mm voxel size, electrical voltage of 120 kVp and 36.9 mAs.

Initially, for sample size estimation, an assumed effect size of 2.0 mm increase in intermolar distance with a standard deviation of 3.0 mm after treatment was considered clinically significant at a 5% significance level and 80% power. Under these assumptions, a minimum of 19 patients were required.

The final sample was collected between 2014 and 2018, totaling 64 CT scans—32 in the pre-treatment phase (T1) and 32 in the post-treatment phase (T2). The patients of both genders and aged between 16 and 53 years old underwent CT scans initially for anatomical assessment and surgical planning for miniplate installation and removal at treatment completion.

Patient records and tomographic images were screened to obtain the sample. The selection criteria for inclusion of patients were: (a) diagnosis of skeletal anterior open bite (SAOB); (b) treatment with four miniplates (two in the posterior region of the maxilla and two in the posterior region of the mandible); (c) patients undergoing the same orthodontic treatment protocol; (d) presence of all permanent teeth; (e) CBCT in the initial and final phases of orthodontic treatment. Exclusion criteria included (a) severe asymmetries, syndromes, cleft lip and palate cleft; (b) history of facial surgeries.

### Sequence of orthodontic treatment

Orthodontic treatment protocol involved Ricketts Standard prescription brackets with 0.018″ × 0.028″ slots (Forestadent®, Pforzheim, Germany) were bonded directly onto the clinical crown center. Two maxillary orthodontic miniplates, in the shape of a “T”, were positioned at the region of the right and left zygomatic crests. Additionally, two mandibular miniplates, also in the shape of a “T”, were placed on the posteroinferior region of the mandibular buccal cortex on both the right and left sides. Alignment and leveling were achieved with progressive increase in wire size including 0.016″ × 0.016″ Neo Sentalloy^®^ 80 g Dentsply GAC International^®^ (Iceland, NY, USA), 0.016″ × 0.016″ Titanol low force^®^ 80 g Forestadent® (Pforzheim, Germany), 0.016″ × 0.022″ Titanol low force® 120 g Forestadent^®^ (Pforzheim, Germany), 0.016″ × 0.016″ Blue Elgiloy^®^ Rocky Mountain Orthodontics^®^ (Denver, CO, USA) e 0.016″ × 0.022″ Blue Elgiloy^®^ Rocky Mountain Orthodontics^®^ (Denver, CO, USA) [13].

The skeletal open bite was treated through posterior intrusion, distalization and subsequent expansion of the maxillary and mandibular posterior teeth. The mechanics that were used for intrusion included the use of elastomeric alloys—Ultra Thread Round Solid (GAC)—with a diameter of 0.030″, with a force intensity between 150 and 200 g, attached from the miniplates to the teeth or to the wire, depending on the desired force vector. For distalization, sliding jigs activated with E-links® Modules TP Orthodontics^®^ (La Porte, IN, USA) elastics with a force of 300–400 g [11–13] were used.

### Image analysis

**Fig. 1:**
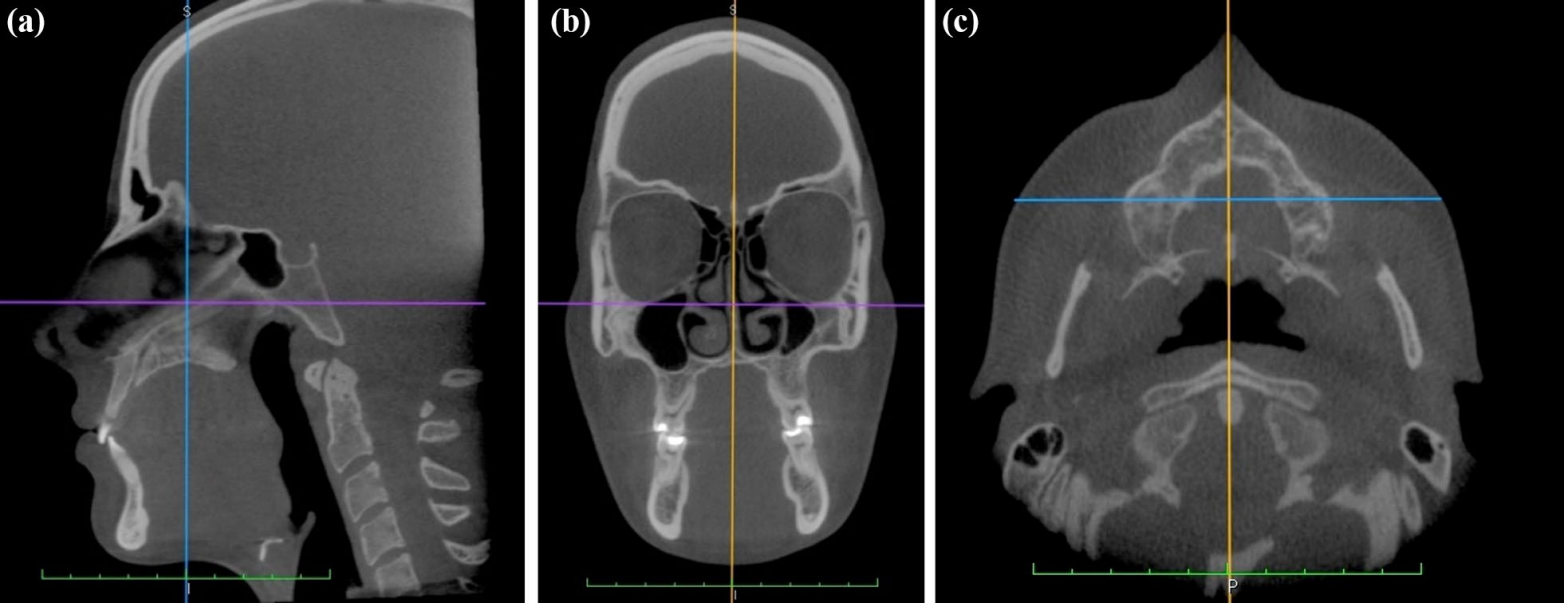
Determination of the reference planes and head positioning. **A** Sagittal section—Frankfurt Axial Plane or Horizontal Plane (purple line) and Coronal Plane (blue line), **B** Coronal section—Axial plane (purple line) and MSP (yellow line). **C** Axial section—MSP (yellow line) and Coronal Plane (blue line)

DICOM files from pre- (T1) and post- (T2) treatment, were imported into Osirix Medical Imaging software (Open-Source, Pixmeo, Geneva, Switzerland) for analysis. Image standardization was performed according to the frankfurt horizontal, mid-sagittal and coronal planes [14]. In the sagittal view, the right orbital and right porion points were identified and positioned to align with the Frankfurt Horizontal plan or axial Plan coinciding with the horizontal line of the software (Fig. 1A). In the coronal view, the right and left orbital points were marked and positioned to align with the horizontal line of the software. Additionally, the midsagittal plane (MSP) was established using reference points including the crista galli (CG), anterior nasal spine (ANS) and basium (Ba), with the midline of the software precisely positioned along the patient's midsagittal plane (Fig. 1).

**Fig. 2:**
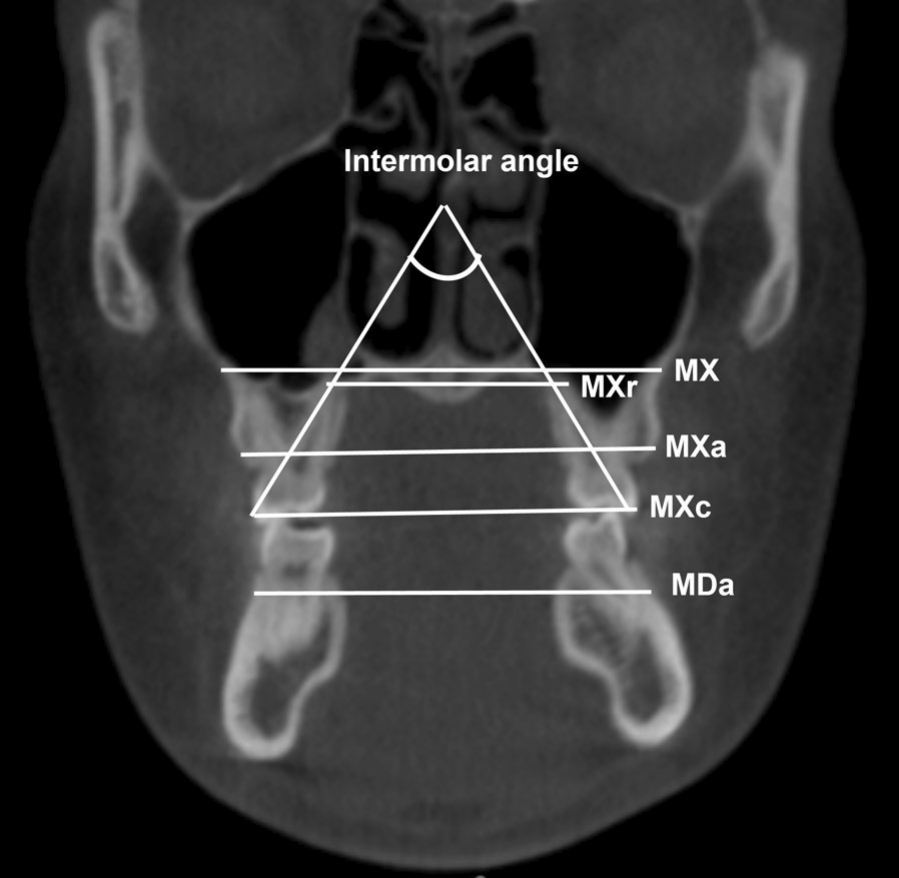
Cone beam computed tomography image in coronal view showing the linear measurements: maxillary base width (MX), maxillary alveolar width (MXa), maxillary root width (MXr), maxillary tooth cusp width (MXc), mandibular alveolar width (MDa) and angular measurement: intermolar angle (IA)

Then, all the measurements were taken in the coronal view. To locate the appropriate coronal view, the upper first molars were positioned in reference to the axial view, where both the right and left palatal roots could be visualized in the same view. So, the most anterior coronal view of both roots was selected for analysis (Fig. 2) [1, 15–17].

#### Linear measurements

- Maxillary base width (MX): The linear distance between the points formed by a straight line touching the lower contour of the nasal cavity and meeting the right and left buccal alveolar contour of the maxilla;
- Maxillary alveolar width (MXa): The distance from the buccal alveolar crest on the right to the left side.
- Maxillary root width (MXr): The distance between the most superior point of the palatal root of the maxillary first permanent molar on both the right and left sides.
- Maxillary dental cusp width (MXc): The distance from the mesiobuccal cusp of the first permanent molar on the right side to the left side.
- Mandibular alveolar width (MDa): The distance between the most convex region of the buccal cortical bone, opposite the furcation of the mandibular first molars [17]; This measurement is made through the distance between the two points, from the right side to the left side.

#### Angular measurement

Intermolar angle (IA): Formed by lines connecting the apex of the palatal root (Mr) to the tip of the buccal cusp (Mc) of the right and left maxillary first molars.

The measurements at each time point (T1 and T2) were conducted separately by a single orthodontist evaluator (EVM), who was trained in the methodology of this study. After 20 day interval, all images were remeasured to assess reproducibility.

### Statistical analysis

The values obtained were treated statistically using the SPSS v. 25 (IBM Corp., SPSS Statistics for Windows, v.25). First, the distribution of data was analyzed using the Shapiro–Wilk Normality Test to confirm normal distribution. Subsequently, the paired t- test was applied to compare the initial and final measurements with a significance level of 5%. To minimize the error, the averages of the first and second measurements were used. For the detection of random error, the Intraclass Correlation was computed, represented by a value ranging from 0 to 1, indicating the degree of reproducibility, with values: < 0.40, indicating poor reproducibility; between 0.40 and 0.75, indicating moderate reproducibility; and > 0.75, indicating high reproducibility. Finally, Pearson correlation analysis was conducted to assess the relationship between the intrusion amount and the maxillary dental cusp width.

## Results

A total of 32 tomographic images were evaluated, which 71.9% from female patients and 28.1% from male patients. The mean age was 33.8 years, with a standard deviation of 12.8 years, ranging from 16 to 53 years. The overbite of SAOB patients ranged from 0 (edge to edge) to − 7.6 mm. To assess the intra-examiner agreement, the differences in the evaluated parameters was observed using the Intraclass Correlation, with a 95% confidence interval, as shown in Table 1. It was noted that all assessed parameters exhibited moderate to high reproducibility, indicating high intra-examiner agreement, except for the maxillary dental cusp and intermolar angle, which demonstrated poor reproducibility (< 0.40), suggesting potential discrepancies between the first and second measurements.

**Table 1:** Intra-examiner agreement on the parameters evaluated in the study

	ICC (95% IC − min/max)	
	T1	T2
Maxillary base width	0.71 (0.48/0.84)	0.77 (0.59/0.88)
Maxillary alveolar width	0.36 (0.01/0.63)	0.83 (0.68/0.91)
Maxillary dental cusp width	0.21 (− 0.14/0.52)	0.36 (0.01/0.62)
Maxillary root width	0.70 (0.46/0.84)	0.78 (0.59/0.88)
Intermolar angles	0.57 (− 0.29/0.39)	0 (− 0.34/0.34)
Mandibular alveolar width	0.48 (0.16/0.71)	0.75 (0.55 / 0.87)

Among the assessed parameters, the maxillary alveolar, dental cusp, and mandibular alveolar widths, as well as the intermolar angle displayed statistically significant differences between the pre and post-treatment phases (*p* < 0.05). However, the widths of the maxillary and root base did not exhibit significant differences (Table 2). There was no significant correlation observed between the amount of intrusion and the maxillary dental cusp width, with the correlation coefficient considered poor (Table 3).

**Table 2:** Paired t test between the parameters evaluated in the study

Parameters	Initial		Final		Difference	*p*
	Mean	SD	Mean	SD		
Maxillary base width (mm)	59.62	3.29	59.81	3.09	0.19	0.809
Maxillary alveolar width (mm)	56.25	2.86	57.94	2.56	1.69	0.015*
Maxillary dental cusp width (mm)	53.43	3.14	56.53	2.70	3.10	< 0.001**
Maxillary root width (mm)	33.87	2.72	33.48	2.47	-0.39	0.549
Intermolar Angles (degrees)	61.41	8.57	72.60	6.95	11.18	< 0.001**
Mandibular alveolar width (mm)	54.20	2.91	56.15	2.65	1.96	0.007**

*Statistically significant difference at the 5% level

**Statistically significant difference at the 1% level

**Table 3:** Pearson correlation analysis between the average intrusion and average width changes

	Average width differences	Average intrusion right first molar	Average intrusion left first molar	Average intrusion (both molars)
	0.34 mm	1.5 mm	1.3 mm	1.4 mm
Pearson r		0.262	0.325	0.382
P (sig)		0.205	0.113	0.060

Correlation was performed between the intrusion amount and the width changes

## Discussion

This study used CBCT images to evaluate, in the transverse dimension, the effects of orthodontic treatment with skeletal anchorage for managing SAOB patients. The present sample consisted of patients who underwent posterior intrusion and distalization using slot 0.018″ appliances. Therefore, this study is the first to quantitatively evaluate transverse changes after treatment using four orthodontic miniplates in patients with anterior open bite.

Regarding the sample size, assumptions were made considering that the intermolar distance would not clinically increase more than 1.0 mm on each side when the arch width is within normal limits. Hence, a net increase of 2.0 mm in arch width was deemed clinically significant. Based on these assumptions, a minimum of 19 patients were required. The present retrospective observational cohort study collected records from 32 patients, ensuring the power of the present study to detect true differences when present.

Analysis of method error revealed poor reproducibility (< 0.40) only for the variables tooth cusp width and intermolar angle, suggesting possible discrepancies between initial and subsequent measurements. Difficulty in marking tooth width points may have arisen due to the presence of restorations causing metallic artifacts in the tomographic image. However, these discrepancies were considered clinically insignificant. Concerning the intermolar angle, difficulty in aligning four reference points on the same section plane may have led to partial presentation of anatomical structures in each coronal section, causing variations in the positioning of measurement reference points. Previous studies have reported similar findings [1, 18].

Changes in the transverse dimension can be part of therapeutic goals through orthopedic or orthodontic management. Orthopedic expansion is indicated to preserve alveolar bone and achieve width increase through the midpalatal suture. Slight differences were observed even after rapid maxillary expansion with appliances like Haas and Hyrax [19]. However, Baratieri et al. [16] found significant increase at the bone and alveolar levels, without changes in molars. Expanders supported on mini-implants (MARPE) tend to produce minimal alveolar effects as they are directly attached to the palatine bone [20]. Dentoalveolar expansion orthodontically may be achieved without buccal alveolar bone increase [21, 22].

In the present study, the width of the maxillary base (Mx) showed slight dimensional changes, akin to those induced by orthopedic expanders in young patients [19]. Additionally, at the alveolar level, the present study showed an increase in the maxillary alveolar width of the maxillary first molars, without bone resorption. These findings corroborate with previous studies that used rapid maxillary expansion appliances (dento-supported, dentomuco-supported, and dento-bone-supported) which reported a greater increase in the buccal inclination of the teeth and the alveolar process compared to orthopedic expansion [1, 19].

It is important to note that buccal expansion was not the primary therapeutic purpose in this study, however, an increase in the width of the dental cusp and the intermolar angle of the maxilla was observed. These findings can be considered as adjunct effect due two factors. One factor may be attributed to distalization, while the other is due to biomechanical effect. The miniplates were placed buccally, away from the center of resistance. When intrusion forces were applied buccally, a tendency for buccal flaring was provoked, which was indeed the most common movement in the intrusive mechanics [23]. Despite this, in the present study, the change in the transverse dimension was independent of the amount of intrusion carried out. According to Consolaro and Furquim [24], the natural inclinations of the teeth may also contribute to these buccal tilting movements under intrusive mechanics. The adjunct effect found in the present study could be necessary if crossbite is present in SAOB patients, but it should be controlled or minimized when the transverse dimension is within normal limits.

Regarding root width, no significant difference was found in the present study. This finding suggests that the expansion effect is restricted only at the crown level. Due to significant intermolar angle changes, buccal tipping likely occurred with the fulcrum at the apex of the molar root. This controlled inclination probably occurred because the molar palatal root may offer greater resistance to movement when intrusion forces were applied [25].

When the occlusal surface is taken as a reference, the Wala border coincides with the most prominent portion of the buccal alveolar bone. Its identification compensates for the lack of a stable anatomical structure to determine the ideal contour of the mandibular arch [26]. In CBCT, the Wala border is located approximately at the border of the cortical bone opposite the furcation of the mandibular first molars, close to the center of tooth resistance (mandibular alveolar width) [17, 26]. In the present study, a significant difference was found in mandibular alveolar width between the first and second measurements, indicating expansion of the mandibular arch. McNamara [27] reported that maxillary arch expansion may promote decompensation of the mandibular arch. In the present study, mandibular alveolar expansion probably occurred due to biomechanical effects similar to those explained for maxillary molars.

The present study focused on the transverse effect of posterior intrusion and distalization mechanics by orthodontics associated with miniplate anchorage in managing SAOB patients. Further studies could investigate transverse effects when using brackets with 0.022″ slots. Additionally, the patients included in this study presented different degrees of SAOB, and consequently different degrees of the intrusive and distalization effect. Therefore, it is suggested more studies include stratified patients.

Regarding the type of anchorage devices, mini-implants, has an advantage over miniplates in terms of the surgical protocol for installation and removal. However, the stability of this approach depends directly on factors such as type, patient age, location and pre-activation healing time [28]. In addition, despite allowing for two-dimensional movement, mini-implants have little transverse effect [29]. Bone anchorage with miniplates, however, allows the force to act with greater intensity and a more uniform distribution, reaching almost the entire length of the maxilla and mandible [5, 13]. Thus, miniplates present more advantages when mechanical demands are of greater magnitude, such as when a larger number of teeth are involved. Therefore, the success of the treatment is closely linked to the mechanics used and knowledge of biomechanics to control bone remodeling and side effects, such as excessive tooth inclination [29, 30]. The relevance of using CBCT images as a research instrument is highlighted, as it allows measurements without overlapping structures. While only tomographic sections in the molar region were used in this study, future studies are recommended to use the superimposition of three-dimensional 3D evaluations of other dental groups [31].

## Conclusions

It could be concluded that posterior intrusion and distalization mechanics with miniplates in the management of skeletal anterior open bite (SAOB) may promote significant expansive changes, evidenced by the increase in molars’ cusp width and buccal inclination, albeit restricted to the alveolar level.

## Data Availability

The data that support the findings of this study are available from Solutions 3D Análise Craniofacial LTDA but restrictions apply to the availability of these data, which were used under license for the current study, and so are not publicly available. Data are however available from the authors upon reasonable request and with permission of Solutions 3D Análise Craniofacial LTDA.

## References

[1] Podesser B, Williams S, Bantleon HP, Imhof H (2004). Quantitation of transverse maxillary dimensions in computed tomography: a methodological and reproducibility study. Eur J Orthod.

[2] LaBlonde B, Vich ML, Edwards P, Kula K, Ghoneima A (2017). Three-dimensional evaluation of alveolar bone changes in response to different rapid palatal expansion activation rates. Dental Press J Orthod.

[3] Garib DG, Raymundo Junior R, Raymundo MV, Raymundo DV, Ferreira SN (2007). Tomografia computadorizada de feixe cônico (cone beam): entendendo este novo método de diagnóstico por imagem com promissora aplicabilidade na Ortodontia. Rev Dental Press Ortod Ortop Facial.

[4] Devanna R (2015). Two-dimensional to three-dimensional: a new three-dimensional cone beam computed tomography cephalometric analysis. J Orthod Res.

[5] Consolaro A (2015). Miniplates and mini-implants: bone remodeling as their biological foundation. Dental Press J Orthod.

[6] Lam R, Goonewardene MS, Allan BP, Sugawara J (2018). Success rates of a skeletal anchorage system in orthodontics: a retrospective analysis. Angle Orthod.

[7] Sousa RLS, Silva E, Portes MIP, Lemos F, Meloti F, Cardoso MA (2021). Transdisciplinary treatments—Orthodontics integrated with oral rehabilitation for the benefit of adult patients: case report. Clin Orthod.

[8] Kawamura J, Park JH, Tamaya N, Oh JH, Chae JM (2022). Biomechanical analysis of the maxillary molar intrusion: a finite element study. Am J Orthod Dentofacial Orthop.

[9] Sugawara J, Kanzaki R, Takahashi I, Nagasaka H, Nanda R (2006). Distal movement of maxillary molars in nongrowing patients with the skeletal anchorage system. Am J Orthod Dentofacial Orthop.

[10] Artese A, Drummond S, Nascimento JM, Artese F (2011). Criteria for diagnosing and treating anterior open bite with stability. Dental Press J Orthod.

[11] Portes MIP, Ertty E, Meloti F, Tien Li A, Ana CFCC, Cardoso MA (2021). Effect of orthodontic maxillary posterior en masse intrusion anchored with miniplates on maxillary sinuses volume. Retrospective CBCT study. J Stomatol Oral Maxillofac Surg..

[12] Silva E, Meloti F, Pinho S, Cardoso MA, Consolaro A (2018). Biomecânica com miniplacas. Rev Clinica Ortod Dent Press.

[13] Santos G, Consolaro A, Meloti F, Cardoso MA, Silva E, Tien Li A, Nascimento MCC (2020). Negligible tooth resorptions after anterior open bite treatment using skeletal anchorage with miniplates. Dental Press J Orthod.

[14] Cevidanes L, Oliveira AE, Motta A, Phillips C, Burke B, Tyndall D (2009). Head orientation in CBCT-generated cephalograms. Angle Orthod.

[15] Pereira JS, Jacob HB, Locks A, Brunetto M, Ribeiro GLU (2017). Evaluation of the rapid and slow maxillary expansion using cone-beam computed tomography: a randomized clinical trial. Dental Press J Orthod.

[16] Baratieri C, Nojima LI, Alves MJ, Souza MMG, Nojima MCG (2010). Transverse effects of rapid maxillary expansion in Class II malocclusion patients: a cone-beam computed tomography study. Dental Press J Orthod.

[17] Tamburrino RK, Boucher NS, Vanarsdall RL, Secchi A (2010). The transverse dimension: diagnosis and relevance to functional occlusion. RWISO J.

[18] Paredes N, Colak O, Sfogliano L, Elkenawy I, Fijany L, Fraser A (2020). Differential assessment of skeletal, alveolar, and dental components induced by microimplant-supported midfacial skeletal expander (MSE), utilizing novel angular measurements from the fulcrum. Prog Orthod.

[19] Garib DG, Henriques JFC, Janson G, Freitas MR, Coelho RA (2005). Rapid maxillary expansion tooth tissue-borne vs. tooth-borne expanders: A CT evaluation of dentoskeletal effects. Angle Orthod.

[20] Carlson C, Sung J, McComb RW, Machado AW, Moon W (2016). Microimplant-assisted rapid palatal expansion appliance to orthopedically correct transverse maxillary deficiency in an adult. Am J Orthod Dentofacial Orthop.

[21] Cattaneo PM, Treccani M, Carlsson K, Thorgeirsson T, Myrda A, Cevidanes LHS, Melsen B (2011). Transversal maxillary dento-alveolar changes in patients treated with active and passive selfligating brackets: a randomized clinical trial using CBCT-scans and digital models. Orthod Craniofac Res.

[22] Basciftci FA, Akin M, Ileri Z, Bayram S (2014). Long-term stability of dentoalveolar, skeletal, and soft tissue changes after non-extraction treatment with a selfligating system. Korean J Orthod.

[23] Apolinário STMPM, Meloti AF, Silva E, Cardoso MA, Consolaro A (2021). Intrusion of posterior teeth using miniplates: intrusive mechanics is not the same as intrusion force. Dental Press J Orthod.

[24] Consolaro A, Furquim L (2011). Mecânica intrusiva gera forças de inclinação e estímulos ortopédicos com reposicionamento dentário e remodelação óssea simultâneos. Dental Press J Orthod.

[25] Sugii MM, Barreto BCF, Vieira-Júnior WF, Simone KRI, Bacchi A, Caldas RA (2018). Extruded upper first molar intrusion: comparison between unilateral and bilateral miniscrew anchorage. Dental Press J Orthod.

[26] Consolaro A, Moura G, Santamaría M (2008). Borda WALA e sua determinação como ponto de referência no tratamento ortodôntico. Dental Press J Orthod.

[27] McNamara JA (2000). Maxillary transverse deficiency. Am J Orthod Dentofacial Orthop.

[28] Yao C-CJ, Lee J-J, Chen H-Y, Chang Z-CJ, Chang H-F, Chen Y-J (2005). Maxillary molar intrusion with fixed appliances and mini-implant anchorage studied in three dimensions. Angle Orthod.

[29] Bento PFL, Macluf-Filho E, Azenha CR, Merhy PM (2020). Tratamento da má oclusão de Classe II com padrão vertical de crescimento com mini-implante na região da crista infrazigomática. Rev Clín Ortod Dental Press.

[30] Andrucioli MCD, Matsumoto MAN (2020). Transverse maxillary deficiency: treatment alternatives in face of early skeletal maturation. Dental Press J Orthod.

[31] Cevidanes LH, Bailey LJ, Tucker GR, Styner MA, Mol A, Phillips CL (2005). Superimposition of 3D cone-beam CT models of orthognathic surgery patients. Dentomaxillofac Radiol.

